# Attitudes towards Potential New Tobacco Control Regulations among U.S. Adults

**DOI:** 10.3390/ijerph15010072

**Published:** 2018-01-05

**Authors:** Allison M. Schmidt, Sarah D. Kowitt, Allison E. Myers, Adam O. Goldstein

**Affiliations:** 1 Department of Health Behavior, Gillings School of Global Public Health, University of North Carolina at Chapel Hill, Chapel Hill, NC 27599, USA; kowitt@email.unc.edu (S.D.K.); aem@countertools.org (A.E.M.); 2 Counter Tools, Carrboro, NC 27510, USA; 3 Lineberger Comprehensive Cancer Center, University of North Carolina at Chapel Hill, Chapel Hill, NC 27599, USA; adam_goldstein@med.unc.edu; 4 Department of Family Medicine, University of North Carolina at Chapel Hill, Chapel Hill, NC 27599, USA

**Keywords:** tobacco control, public opinion, smoking, attitudes

## Abstract

Favorable attitudes towards tobacco control policies can facilitate their implementation and success. We examined attitudes toward four potential U.S. Federal tobacco regulations (banning menthol from cigarettes, reducing nicotine levels in cigarettes, banning candy and fruit flavored electronic cigarettes, and banning candy and fruit flavored little cigars and cigarillos) and associations with individual and state variables. A nationally representative phone survey of 4337 adults assessed attitudes toward potential policies. Weighted logistic regression was used to assess relationships between attitudes and demographic factors, smoking behavior, beliefs about the government (knowledge, trust, and credibility), exposure to tobacco control campaigns, and state variables from the US Centers for Disease Control and Prevention (CDC) State Tobacco Activities Tracking and Evaluation (STATE) System. Most respondents supported three out of four policies. Respondents that were female, non-white, Latino, living below the poverty line, had less than high school education, were of older age, did not smoke, had higher trust in government, and were exposed to national tobacco control campaigns had higher odds of expressing favorable attitudes toward potential new tobacco regulations than did their counterparts. No state-level effects were found. While differences in attitudes were observed by individual demographic characteristics, behaviors, and beliefs, a majority of participants supported most of the potential new tobacco regulations surveyed.

## 1. Introduction

In the US, the Food and Drug Administration (FDA), under the Family Smoking Prevention and Tobacco Control Act (FSPTCA) of 2009, has the authority to regulate the manufacture, marketing, and sales of tobacco products, and communicate the risks of using tobacco to the American public [1]. Tobacco control policies and regulations are some of the most effective types of interventions to prevent and reduce tobacco use [2,3]. In the past decade, the FDA has banned flavored cigarettes; established restrictions on youth access; increased restrictions of ‘light’, ‘mild’, or ‘low tar’ descriptors on tobacco products; required revised health warnings on smokeless tobacco products [4]; and most recently asserted its authority to regulate all products derived from tobacco, including electronic cigarettes (e-cigarettes) [5]. Policies that involve regulating the contents and flavors of other tobacco products, particularly with expanded FDA authority under ‘deeming’ regulations [5] would be in the FDA’s purview of available regulations. Examples of key policies the FDA has expressed interest in considering that are likely to have wide population impact include reducing nicotine [6] or regulating menthol [7] in cigarettes, or banning candy and fruit flavored e-cigarettes and little cigars [8]. To date, little research exists about potential attitudes of U.S. adults to these policies and factors associated with these attitudes.

Favorable attitudes towards tobacco control policies can contribute to policy adoption [9], effective implementation [10], and success in changing tobacco-related attitudes and behaviors [11,12]. Understanding attitudes towards policies and regulations can help gauge reception of these policies and potential reactions to them. Such research is especially timely for policies the FDA is currently considering, including lowering levels of nicotine in cigarettes, as announced in a recent FDA statement, about which they plan to solicit data and public comment soon [13]. Additionally, local, state, and national policymakers can implement related restrictions on tobacco products, in which public support plays a role as well [14]. Further, understanding groups that are more likely to support certain policies can suggest target audiences for communication campaigns that can encourage support for and compliance with new policies [15,16]. Thus, assessing attitudes towards potential tobacco control policies, and factors associated with such attitudes, have implications for policymakers and tobacco control advocates.

Past research has identified individual level factors associated with attitudes towards tobacco prevention and control. Across several studies, women (compared to men), non-White individuals (compared to White individuals), non-Hispanic individuals (compared to Hispanic individuals), and older individuals have been found to be more supportive of some tobacco control policies [17,18,19,20,21]. There have been more mixed findings about educational attainment; in some studies, lower educational attainment has been positively associated with supportive policy attitudes [18,21] although other studies have found no association [17], or contrary findings [20,22]. Smoking behavior has been consistently associated with policy attitudes such that nonsmokers tend to hold more favorable attitudes towards tobacco control policies than smokers [17,18,19,20,21,23]. With respect to beliefs, political ideology may impact attitudes, such that favorable attitudes towards tobacco control decreased as individuals showed more conservative political views and had less knowledge of tobacco’s health impacts. Additionally, trust in the government has been shown to be positively associated with support for tobacco control legislation. Knowledge about, perceived credibility of, and trust in regulatory agencies may also affect attitudes towards such agencies’ policies, although this has yet to be explicitly tested. 

State-level factors, which may indicate a stronger or weaker tobacco control environment, may also influence policy attitudes, although research in this area is limited. One study did not find any significant effects of state-level contextual variables—including state compliance with youth access restrictions, taxes on tobacco, strength of smokefree policies, and whether the state is tobacco producing—on attitudes towards tobacco point of sale policies [17]. However, relatively few studies have employed such a multi-level framework in their examination of attitudes toward tobacco control policies, a gap the current research aims to help fill.

The current research examines attitudes toward four potential new Federal tobacco regulations (banning menthol from cigarettes, reducing nicotine levels in cigarettes, banning candy and fruit flavored electronic cigarettes, and banning candy and fruit flavored little cigars and cigarillos) and associations of such attitudes with several factors. Previous research has measured attitudes towards various tobacco control measures, including smoke-free policies [19,20,23,24], point of sale policies [17,25], raising the age of tobacco use [26], banning menthol in cigarettes [18,21,27], reducing nicotine content in cigarettes [28], and banning flavored e-cigarettes [29,30]. The current research extends previous work by simultaneously assessing a range of factors, including trust in government, knowledge and credibility beliefs about the FDA, exposure to tobacco control communication campaigns, and state-level variables that may influence attitudes across a diverse group of potential new tobacco control policies, using a nationally representative sample of U.S. adults. 

## 2. Materials and Methods 

### 2.1. Data Sources 

Data utilized in this research come from two sources: (1) a nationally representative telephone survey administered by the Center for Regulatory Research on Tobacco Communication (CRRTC) between 15 September 2014 and 31 May 2015; and (2) a set of state-level variables retrieved from national public health agency tracking systems. The CRRTC phone survey included questions on tobacco regulatory constructs, including tobacco product use, tobacco constituent perceptions, and tobacco regulatory agency credibility in a nationally representative sample of US adults. In order to ensure adequate representation among smokers and young adults, high-smoking/low-income areas were oversampled, a method that has been implemented in previous nationally representative surveys. More details on the sampling and data collection procedures are described in Boynton et al. (2016) [31].

State-level factors were retrieved from the Centers for Disease Control and Prevention’s (CDC) State Tobacco Activities Tracking and Evaluation (STATE) System, a database of publicly accessible state-level tobacco-related data [32]. State-level data (smoking prevalence and cigarette excise tax rates) were merged into the national survey data based on each individual’s state residence at the time of the survey. 

### 2.2. Measures

#### 2.2.1. Policy Attitudes

Attitudes towards four different potential new tobacco regulations were measured with the items: (1) “Do you think the FDA should ban menthol, a minty flavor, from cigarettes?”; (2) “Do you think the FDA should require companies to reduce the nicotine level in cigarettes?”; (3) “Do you think the FDA should ban candy and fruit flavored e-cigarettes and other vaping devices?”; and (4) “Do you think the FDA should ban candy and fruit flavored little cigars and cigarillos?” Participants were given response options of “yes” and “no”, although if they responded that they did not know how they felt about these policies, “don’t know” responses were coded as such. For analyses, response options were dichotomized as: “yes” vs. “no” or “don’t know” to test the odds of an affirmative “yes” policy support response. “Don’t know” was not explicitly offered to participants as a response option.

#### 2.2.2. Individual Factors

Individual demographic characteristics included gender, race (white or non-white), ethnicity (Latino or non-Latino), age (continuous), living below the poverty line, and having a less than high school education. With respect to smoking behavior, individuals were classified as current smokers if they reported both having smoked at least 100 lifetime cigarettes and were currently smoking every day or some days.

Individual knowledge and beliefs included trust in government, knowledge of FDA regulation of tobacco products, and perceived credibility of the FDA as a tobacco regulator. Trust in government was measured by one item: “How much trust do you have in the Federal government?” Responses were coded as 0 = none at all, 1 = not very much, 2 = no opinion, 3 = a fair amount, 4 = a great deal; participants who reported “don’t know” were coded as missing. Knowledge of FDA regulation of tobacco products responses was measured with three items asking participants if they have heard of the FDA, and if they think the FDA regulates how cigarettes are made, advertised, or sold in stores. Responses were coded as “yes” or “no/don’t know” and summed to create an index ranging from 0 to 3, with higher values indicating more knowledge of FDA regulation of tobacco products. Perceived credibility of the FDA as a tobacco regulator was measured with eight positively-worded items asking whether participants found the FDA trustworthy and expert at regulating tobacco products. Responses to each item were “yes”, “no”, and “don’t know” (not offered to participants but coded if expressed). Affirmative “yes” responses were summed to create a scale ranging from 0 to 8, with higher values indicating higher perceived FDA credibility. 

Media exposure was measured with one item per survey respondent. Each respondent was randomly assigned to hear one of two questions: whether they had, “ever seen or heard any ads on television or radio with the slogan, ‘Tips from a Former Smoker’ or ‘The Real Cost’” (sponsored by the CDC and FDA respectively, although this was not communicated to participants). Affirmative “yes” responses were coded as “1”. Both of these campaigns educate smokers and nonsmokers about the costs and consequences of smoking and are widely aired during a variety of television and radio programs. “Tips” advertisements (running 2012–present) focus on a range of health consequences due to smoking from the perspective of those who have experienced them [33]. The goal of these advertisements is to promote broad awareness of the negative health effects of tobacco use and promote quitting among smokers. “The Real Cost” (running 2014–present) targets young people to prevent smoking by teaching about the health and other negative effects of tobacco use and addiction [34]. 

#### 2.2.3. State-Level Factors 

State-level variables from the CDC’s STATE System included adult smoking prevalence and cigarette excise tax rates. State prevalence of adult smoking in 2014 was matched by state residence to the survey data. State cigarette excise tax rates were measured quarterly and matched to the national survey data on state, year, and quarter by survey date. Given the non-linear distribution of tax rates, three tax rate tertiles were created to denote low, medium, and high excise tax rates. 

### 2.3. Data Analysis

Out of the sample of 5014 individuals, 677 were excluded because they either had missing data on one or more key variables, or reported not having heard of the FDA. For consistency across models, only individuals with complete data across all relevant variables were included in the analyses (N = 4337). We used SAS version 9.4 (SAS Institute Inc., Cary, NC, USA) survey procedures to account for the complex survey design and sampling weights [35]. Weighted logistic regression was used to test the odds of reporting favorable attitudes towards each of the potential FDA tobacco regulations measured. We set critical α = 0.05 and used two-tailed statistical tests.

## 3. Results

### 3.1. Sample Characteristics

Weighted sample characteristics are shown in Table 1. The sample was predominantly non-Latino (12.8% Latino), and White (73.0%), and about one-sixth were current smokers (17.6%). Mean values for trust in the government were 1.99 (SD: 0.03) on a scale of 0 to 4. Mean levels of knowledge about the FDA’s role as a tobacco regulator were relatively low at 1.35 (SD: 0.03) on a scale of 0 to 3, and mean views of FDA credibility as a tobacco regulator were moderate at 4.67 (SD: 0.06) on a scale of 0 to 8. About one-third of the adult sample (36.7%) reported being exposed to either the CDC’s or FDA’s national tobacco control communication campaigns.

Respondents showed generally favorable attitudes toward four potential new Federal tobacco regulations (Table 2). A majority of respondents supported regulations to reduce nicotine in cigarettes (71.0%), ban candy or fruit flavored little cigars (54.4%), or ban candy or fruit flavored e-cigarettes (56.4%). Only a third of respondents (33.5%) supported a ban on menthol in cigarettes. 

### 3.2. Variable Associations with Attitudes towards Potential New Tobacco Regulations

#### 3.2.1. Attitudes towards Banning Menthol in Cigarettes 

Variables associated with higher odds of reporting favorable attitudes toward banning menthol in cigarettes included being non-white (AOR 1.43; 95% CI: 1.08, 1.88), having a less than high school education (AOR 1.51; 95% CI: 1.15, 1.97), and being a nonsmoker (AOR 2.21; 95% CI: 1.59, 3.08). 

#### 3.2.2. Attitudes towards Reducing Nicotine in Cigarettes

Variables associated with higher odds of reporting favorable attitudes toward reducing nicotine in cigarettes included being female (AOR 2.14; 95% CI: 1.66, 2.78), being non-white (AOR 1.68; 95% CI: 1.18, 2.40), being Latino (AOR 1.88; 95% CI: 1.26, 2.81), being of older age (AOR 1.01; 95% CI: 1.01, 1.02), and being a non-smoker (AOR 1.76; 95% CI: 1.30, 2.39) (Table 3). 

#### 3.2.3. Attitudes towards Banning Candy/Fruit Flavors in E-Cigarettes

Variables associated with higher odds of reporting favorable attitudes toward banning candy and fruit flavored e-cigarettes included being female (AOR 1.67; 95% CI: 1.34, 2.08), being non-white (AOR 1.39; 95% CI: 1.07, 1.79), living below the poverty line (AOR 1.46; 95% CI: 1.01, 2.11), having less than a high school education (AOR 1.30; 95% CI: 1.01, 1.66), being of older age (AOR 1.03; 95% CI: 1.03, 1.04), being a nonsmoker (AOR 2.04; 95% CI: 1.53, 2.73), having higher the trust in government (AOR 1.21; 95% CI: 1.10, 1.34), and being exposed to a national tobacco control campaign (AOR 1.35; 95% CI:1.09, 1.68).

#### 3.2.4. Attitudes towards Banning Candy/Fruit Flavors in Cigarillos and Little Cigars

Variables associated with higher odds of reporting favorable attitudes toward endorsing a ban on candy and fruit flavored little cigars and cigarillos included being female (AOR 1.84; 95% CI: 1.47, 2.30), being non-white (AOR 1.49; 95% CI: 1.15, 1.95), living below the poverty line (AOR 1.48; 95% CI: 1.01, 2.16), being of older age (AOR 1.03; 95% CI: 1.02, 1.04), being a nonsmoker (AOR 2.06; 95% CI: 1.54, 2.77), having higher trust in the federal government (AOR 1.18; 95% CI: 1.07, 1.30), and being exposed to a national tobacco control campaign (AOR 1.48; 95% CI: 1.18, 1.84). 

## 4. Discussion

This is the first nationally representative sample of U.S. adults to simultaneously examine not only attitudes toward potential new Federal tobacco regulations, but also associations between multiple relevant variables and attitudes towards these regulations. The findings have three implications for researchers, practitioners and policy-makers. First, consistent with past research, the majority of US adults had favorable attitudes towards policies that would reduce nicotine in tobacco products, ban candy and fruit flavors in e-cigarettes, and ban candy and fruit flavors in little cigars and cigarillos [17,18,21,28]. The current study found relatively more of our sample held favorable attitudes towards banning candy and fruit flavors than has been found in some past research [29,30]. Individuals living below the poverty line, a group that has the highest rate of smoking [36], were more likely to support policies banning candy and fruit flavors than their counterparts living above the poverty line. Our results are consistent with other studies finding individuals with lower income may be more supportive of tobacco industry regulations [37]. In our study, non-white individuals were also more supportive of tobacco control regulations, consistent with findings in previous research [17]. Both of these groups are frequently targets of tobacco company marketing and promotions [38,39]. Our results show that these vulnerable groups, who are likely to be the most impacted by tobacco regulations that could help reduce disparities in tobacco use, are generally supportive of these new policies, an important finding for tobacco control advocates to know and share. Having evidence that certain demographic and behavioral groups have more favorable attitudes towards potential new tobacco regulations from a national sample of US adults is useful for policymakers and organizations to more fully understand public views on, and to plan communication and other efforts around, these policy issues.

Second, supportive attitudes towards banning menthol in cigarettes was found to be mixed [17,18] although higher than in at least one other study [21]. This may be in part because people do not associate menthol use with youth smoking, when it does play a significant role in youth smoking initiation and smoking progression [40]. Public support for tobacco control is often highest around initiatives to prevent youth access to tobacco [17]. Additionally, as the majority of users of menthol cigarettes in the U.S. are African American, in part due to targeted marketing by the tobacco industry [41], banning menthol is seen by some as a racialized issue with potential unintended consequences for African American communities, views also promoted by tobacco companies themselves [42]. (However, at least one study has found African American smokers to be more supportive of banning menthol than non-African American smokers [43].) While banning menthol in cigarettes was actively supported by about one-third of our sample, nearly one-fifth of our sample reported not knowing whether they supported this policy or not, consistent with past research finding ambivalent or “don’t know” responses around this issue [21]. This may suggest an area for education about the benefits and implications of banning menthol, especially if people do not know about its role in youth smoking initiation, its association with disease risk given its more mild flavor, or its association with higher nicotine dependence and reduced cessation success [7]. Further understanding these “don’t know” responses, while outside the aim of the current study, would be a valuable aim of future research to guide educational interventions.

Third, multiple variables seem related to policy support of new potential Federal tobacco regulations. For instance, exposure to tobacco communication campaigns had stronger associations with attitudes than did state-level factors. With respect to communication, having reported exposure to tobacco control communication campaigns increased the odds of favorable attitudes towards tobacco regulations. While not tested in this research, exposure to an anti-tobacco communication campaign may enhance favorable attitudes towards tobacco control. In two of our models, exposure to a mass media campaign to prevent and reduce tobacco use (either the CDC’s “Tips from Former Smokers” campaign or the FDA’s “The Real Cost” campaign), were associated with attitudes supporting tobacco regulation. Several national campaigns like these already exist, and the FDA is expanding its presence and reach to multiple target populations [34,44,45]. Future research could test whether exposure to messages about a specific tobacco control policy influence attitudes towards these policies, as some past work has found [46,47]. 

Literature on social norms suggests that favorable attitudes may be increased also if individuals perceive that other members of the public have supportive attitudes towards tobacco regulation and believe that such policies fulfill a greater social good. Overall, while policymakers should strive to enact evidence-based tobacco control policies, even in face of divided public attitudes, understanding the landscape of public attitudes and what can be done to change them is important.

### Strengths and Limitations

A key strength of this study includes its synthesis of individual and environmental level data from a nationally representative sample of U.S. adults and a national public health agency tracking system. Study findings may be limited by the use of a single-item split-panel measure to assess media exposure, one of our key findings, which was measured differently for half of the respondents (as half were asked about recalling a campaign from the CDC and half from the FDA). In addition, recall bias may have affected responses; individuals who were exposed to such campaigns, and recalled their exposure, may have been qualitatively different than individuals who did not see or recall such messages, although we were not able to discern whether or not this was the case. Additionally, we do not know why participants responded the way they did to different potential policies (for example, it is unclear if people know nicotine is the addictive agent in cigarettes, or just a harmful chemical). Further research on the public’s awareness and understanding of tobacco control regulations, potential reactance, or resistance, to such policies, and intensity of attitudes or support (e.g., weaker or stronger support), would enhance our understanding of these responses. 

## 5. Conclusions 

Overall, a majority of the U.S. public showed support for most of these new potential tobacco control policies that have the potential to reduce disparities in tobacco use. We identified the influence of individual demographic characteristics, smoking behavior, and beliefs on shaping attitudes towards tobacco control policies. Several demographic characteristics were consistently associated with support for tobacco control policies, as was exposure to messages designed to prevent and reduce smoking, with implications for enhancing favorable policy attitudes.

## Figures and Tables

**Table 1:** Weighted demographic characteristics of respondent sample (N = 4337).

	N	%	Mean	SE
**Gender**				
Female	2164	51.1		
Male	2069	48.9		
**Race**				
White	2980	73.0		
Non-white	1252	29.6		
**Latino**	543	12.8		
**Living below poverty line**	680	16.1		
**<HS Education**	1692	40.0		
**Age** (Range: 18–95 years of age)			47.02	0.53
**Smoking Behavior**				
Smoker	746	17.6		
Nonsmoker	3487	82.4		
**Trust in gov’t** (Scale: 0 to 5)			1.99	0.03
**Knowledge of FDA as tobacco regulator** (Scale: 0 to 3)			1.35	0.03
**Credibility of FDA as tobacco regulator** (Scale: 0 to 8)			4.67	0.06
**Exposed to tobacco control messages**	1555	36.7		
**State adult smoking prevalence** (Range: 9.7–26.7%)			17.5	0.08
**State cigarette excise tax** (Range: $0.17–$4.35)				
Low	808	19.1		
Medium	1370	32.4		
High	2054	48.5		

**Table 2:** Weighted frequencies of responses to “Do you think the FDA should …” (N = 4337).

	Yes	No	Don’t Know
	N (%)	N (%)	N (%)
Ban menthol from cigarettes?	1416 (33.5%)	2083 (61.9%)	734 (17.3%)
Reduce nicotine in cigarettes?	3005 (71.0%)	1040 (24.6%)	188 (4.4%)
Ban candy and fruit-flavored e-cigarettes?	2305 (54.4%)	1748 (41.3%)	180 (4.3%)
Ban candy and fruit-flavored little cigars/cigarillos?	2388 (56.4%)	1682 (39.7%)	163 (3.8%)

**Table 3:** Adjusted odds of support for potential FDA tobacco regulations (N = 4337).

	Ban Menthol in Cigarettes			Reduce Nicotine in Cigarettes			Ban Candy/FF E-Cigs			Ban Candy/FF LCC		
	AOR	95% CI		AOR	95% CI		AOR	95% CI		AOR	95% CI	
**Individual**												
Female	1.26	1.00	1.60	2.14 *	1.66	2.78	1.67 *	1.34	2.08	1.84 *	1.47	2.30
Non-white	1.43 *	1.08	1.88	1.68 *	1.18	2.40	1.39 *	1.07	1.79	1.49 *	1.15	1.95
Latino	1.04	0.72	1.51	1.88 *	1.26	2.81	1.13	0.81	1.58	1.15	0.81	1.65
Living below poverty line	1.21	0.84	1.75	1.35	0.83	2.20	1.46 *	1.01	2.11	1.48 *	1.01	2.16
<HS Education	1.51 *	1.15	1.97	1.24	0.94	1.65	1.30 *	1.01	1.66	1.15	0.89	1.49
Age	1.01	1.00	1.01	1.01 *	1.01	1.02	1.03 *	1.03	1.04	1.03 *	1.02	1.04
Nonsmoker	2.21 *	1.59	3.08	1.76 *	1.30	2.39	2.04 *	1.53	2.73	2.06 *	1.54	2.77
Trust in gov’t	1.12	1.00	1.25	1.12	1.00	1.26	1.21 *	1.10	1.34	1.18 *	1.07	1.30
FDA knowledge	1.01	0.90	1.14	1.09	0.97	1.24	1.01	0.90	1.13	1.01	0.90	1.13
FDA credibility	0.96	0.91	1.01	1.01	0.96	1.07	0.98	0.93	1.03	0.98	0.93	1.03
Exposure to tobacco control campaign	1.12	0.87	1.43	1.31	0.99	1.74	1.35 *	1.09	1.68	1.48 *	1.18	1.84
**State-level**												
State smoking prevalence	0.96	0.93	1.00	1.02	0.98	1.05	0.98	0.95	1.02	0.97	0.94	1.00
State cig. tax	0.96	0.81	1.13	1.05	0.87	1.27	0.90	0.78	1.04	0.86	0.74	1.00

* Denotes significant effect: 95% Confidence Interval does not include 1.
